# The morphology of the proximal femur in cementless short-stem total hip arthroplasty: No negative effect on offset reconstruction, leg length difference and implant positioning

**DOI:** 10.1186/s13018-021-02876-7

**Published:** 2021-12-20

**Authors:** Matthias Luger, Sandra Feldler, Antonio Klasan, Tobias Gotterbarm, Clemens Schopper

**Affiliations:** 1 Department for Orthopedics and Traumatology, Kepler University Hospital GmbH, Krankenhausstrasse 9, 4020 Linz, Austria; 2 Johannes Kepler University Linz, Altenberger Strasse 69, 4040 Linz, Austria; 3 Kepler University Hospital Linz, Krankenhausstrasse 9, 4020 Linz, Austria

**Keywords:** Short stem, Total hip arthroplasty, Dorr classification, Canal flare index, Leg length difference, Offset reconstruction

## Abstract

**Background:**

Correct reconstruction of hip offset (HO) and leg length are important for clinical–functional outcome and patient satisfaction in total hip arthroplasty (THA). The morphology of the proximal femur can pose a risk for increased leg length difference (LLD) in cementless straight-stem THA. We therefore wanted to evaluate, if this is also applicable in THA with a cementless meta-diaphyseal short stem.

**Methods:**

In a retrospective study, 106 patients (index surgery 2014–2019) with unilateral THA and a morphologically healthy hip as a reference (Kellgren–Lawrence ≤ 1) were included. The same cementless short stem with meta-diaphyseal fixation and cementless press-fit cup was implanted. The proximal femur was rated by Dorr’s classification, and subgroups were formed afterward. Measurements were carried out on preoperative and 3 months postoperative anterior–posterior radiographs of the pelvis. Kruskal–Wallis test, Fisher’s exact test and binary logistic regression were performed to evaluate the influence of the anatomical shape on postoperative leg length difference and offset reconstruction.

**Results:**

The Dorr type did not show any significance influence on LLD (*p* = 0.532), or postoperative difference in femoral offset (*p* = 0.243), acetabular offset (*p* = 0.106) and hip offset (*p* = 0.698). Stem alignment (*p* = 0.705) and canal fill indices (CFI I: *p* = 0.321; CFI II: *p* = 0.411; CFI III: *p* = 0.478) were also without significant differences. Logistic regression did not show any significant increased risk for a LLD ≥ 5 mm or ≥ 10 mm as well as HO ≥ 5 mm or ≥ 10 mm.

**Conclusion:**

Reconstruction of hip offset and postoperative leg length difference is not negatively influenced by Dorr type, canal flare index, cortical index and canal-to-calcar ratio in cementless short-stem THA. Implant positioning and canal fill are also not negatively affected by the anatomical shape of the proximal femur.

*Level of evidence*: Level IV.

## Introduction

Total hip arthroplasty (THA) is a very successful and cost-effective surgical management of patients with end-stage osteoarthritis of the hip [1, 2]. Correct reconstruction of the hip geometry is essential in THA in order to avoid adverse outcomes such as impingement and dislocation [3, 4], early implant failure [5], abductor weakness [4, 6] and leg length discrepancy [7]. Accurate reconstruction of hip offset (HO) and leg length demonstrated an additive effect on postoperative clinical outcome [8]. Patients with accurate to slightly increased HO combined with balanced leg length show higher increases in delta Harris hip score (HHS) [8]. A postoperative leg length difference (LLD) and difference in HO above 5 mm are additionally associated with altered gait kinematics [9].

The anatomical shape of the proximal femur can have a significant impact on postoperative LLD and osseointegration of cementless THA [10]. LLD in cementless straight-stem THA depends on the used implant and its fixation [10]. Dorr type A femurs according to Dorr’s classification [11] are 30% more likely to gain more than 5 mm of leg length compared to Dorr type B or C femurs [12]. A higher canal flare index (CFI) corresponded with an odds ratio of 4.5 in cementless femoral stems with metaphyseal fixation for postoperative LLD ≥ 5 mm, while cementless diaphyseal fixation or cemented stems did not show an increased risk [13].

Cementless short stems have been increasingly used in recent years parallel with the use of minimally invasive approaches [14, 15]. Short stems allow an accurate reconstruction of hip offset while keeping LLD at a minimum [16]. While the influence of the morphology of the proximal femur has been evaluated in previous studies, the influence in cementless short-stem THA has not been fully evaluated. Therefore, we conducted this study to evaluate the influence of the proximal femoral morphology on offset reconstruction, leg length difference and implant positioning in THA with a cementless short stem with meta-diaphyseal fixation.

## Methods

### Patients

This retrospective radiological comparative study includes patients of a consecutive series of THAs with the same cementless curved short stem (Fitmore® stem, ZimmerBiomet, Warsaw, IN, USA) and bi-hemispherical press-fit acetabular cup (Allofit®/-S, ZimmerBiomet, Warsaw, IN, USA) performed via a minimally invasive supine anterolateral approach. Fitmore® hip stem is a titanium alloy stem (Ti Al6V4) that has a porolock Ti-VPS coating in the proximal part to enhance bone ingrowth and is available in four different neck angle options (127°, 129°, 137°, 140°) and 14 different sizes (size 1–14) for each offset option [14]. A cementless titanium press-fit cup with or without screws (Allofit®/-S, ZimmerBiomet, Warsaw, IN, USA) was used in all patients. Fitmore® hip stem is available in four different offset options, and therefore, the stem allows an offset reconstruction independent of stem size with superior balance of soft tissue of the hip [17]. The curved design of Fitmore® stem is designed to transmit load proximally and thus to give an optimal fit in the calcar region [18]. The stem has a triple-tapered design to achieve press-fit fixation at the metaphyseal/diaphyseal level and according to the recommended femoral neck resection level [19].

**Fig. 1:**
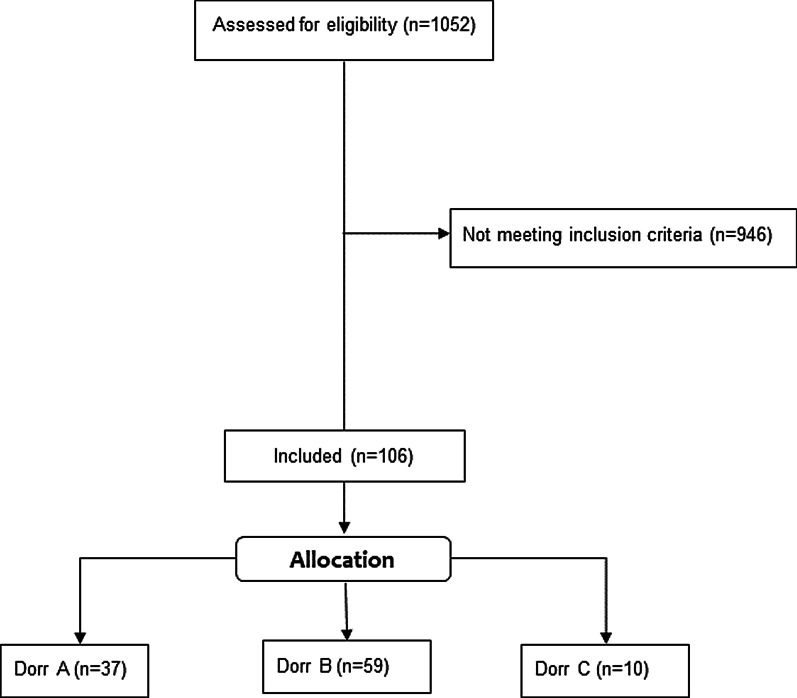
Consort diagram

A consecutive series of 1052 hips in 982 patients with index surgery between 2014 and 2019 were screened for inclusion, and the medical records until 90 days postoperative were evaluated. The preoperative X-rays of the pelvis (both hips in comparison, anterior–posterior view, standing upright) were screened for unilateral THA. Diagnoses for inclusion were primary osteoarthritis, avascular necrosis of the femoral head or mild dysplasia of the hip (Crowe I) [20]. Exclusion criteria were defined as bilateral hip disease (Kellgren–Lawrence > grade 1) [21], a history of prior hip surgery, previous trauma, postoperative complication, reoperation or revision for any reason as well as missing pre- or postoperative radiographs. In total, 106 patients met the inclusion criteria (see Fig. 1). The included patients were then reviewed independently by two reviewers (M.L. and C.S.), who were not involved in the index surgery. The anatomical shape of the proximal femur was determined according to the Dorr classification [11]. In case of different determination of Dorr types between both reviewers, the preoperative X-ray was evaluated together and a consensus agreement was found.

Radiographic measurements were performed on pre- and 3 months postoperative low-centered anteroposterior (AP) radiographs of the pelvis in both groups. Preoperative age at operation, gender, body mass index (BMI) and laterality were recorded. The patient demographics are shown in Table 1.

**Table 1:** Patient demographics, pre- and postoperative measurements

	Dorr A (*n* = 37)	Dorr B (*n* = 59)	Dorr C (*n* = 10)	*P* value
Gender (m/f)	23:14	18:41	0:10	**< 0.001**
Age (in years)	55.3 ± 10.6	58.5 ± 11.3	55.5 ± 12.0	0.163
Side (l/r)	18:19	25:34	6:4	0.547
BMI (kg/m^2^)	29.0 ± 5.4	26.9 ± 4.5	26.7 ± 4.9	0.161
Preoperative measurements				
FO difference (mm)	1 ± 4.2	2.3 ± 3.9	3.3 ± 4.6	0.157
AO difference (mm)	0.4 ± 2.4	0.9 ± 4.1	1.9 ± 1.7	0.209
HO difference (mm)	0.6 ± 3.8	1.4 ± 4.1	4.1 ± 4.8	0.573
LLD (mm)	− 4.5 ± 5.5	− 3.5 ± 4.4	− 3 ± 4.3	0.520
CCD angle (°)	129.8 ± 5.5	131.3 ± 6.4	138.1 ± 7.1	**0.007**
Canal flare index	4.3 ± 0.6	3.8 ± 0.5	3.1 ± 0.4	**< 0.001**
Cortical index	0.64 ± 0.04	0.59 ± 0.03	0.51 ± 0.05	**< 0.001**
Canal-to-calcar ratio	0.54 ± 0.08	0.58 ± 0.05	0.68 ± 0.05	**< 0.001**
Postoperative measurements				
FO difference (mm)	5.3 ± 6.4	7.1 ± 6.6	9.7 ± 6.9	0.243
AO difference (mm)	− 2.7 ± 4.9	− 4.2 ± 4.2	− 5.6 ± 3.6	0.106
HO difference (mm)	2.6 ± 4.9	2.9 ± 5.4	4.1 ± 7.5	0.698
LLD (mm)	− 0.38 ± 5	− 0.31 ± 5.2	1.6 ± 4.9	0.532
Stem alignment (°)	4.9 ± 3	4.5 ± 3.1	4.3 ± 3.4	0.705
Canal Fill Index I (%)	76.7 ± 6.5	78.9 ± 6	78.6 ± 7.6	0.321
Canal fill index II (%)	79.6 ± 7.3	81.5 ± 5.8	80.6 ± 10.5	0.411
Canal fill index III (%)	84.6 ± 9.2	83.5 ± 7.4	78.3 ± 12.9	0.478

Bold values signal statistically significant values in testing

The study was approved by the institutional review board (EK-No.: 1239/2019). Due to the retrospective study design with evaluation of pre-existing medical records, an informed consent was not required. All procedures performed in studies involving human participants were in accordance with the ethical standards of the institutional and/or national research committee and with the 1964 Helsinki declaration and its later amendments or comparable ethical standards.

### Surgical technique and treatment protocol

Surgical procedures were carried out at the author’s institution by surgeons with different levels of experience including 11 consultants and 7 residents. All consultants perform more than 50, all senior consultants more than 100 arthroplasties per year. Resident surgeries were done under the guidance of a consultant. In all cases, a minimally invasive anterolateral Watson–Jones approach in supine position on a standard operating table under laminar airflow was performed. Extremity preparation was performed with threefold antiseptic scrub with alcohol disinfectant. Draping with a sterile adhesive surgical iodine film was used. The skin incision was centered over the greater trochanter. An incision at the border between the tensor fasciae latae and the tractus iliotibialis was performed. Then, the Watson–Jones interval between tensor fasciae latae and gluteus medius was bluntly dissected. A capsulectomy was performed in every case. The standardized peri- and postoperative protocol was identical in all cases, including single-shot antibiotics (Cefuroxime 1.5 g i.v. directly preoperative), weight-bearing as tolerated from the first postoperative day on, Indomethacin 75 mg daily for the prevention of heterotopic ossification on day 1–4 postoperatively and 40 mg low-molecular weight heparin or Rivaroxaban 10 mg for 28 days postoperatively as venous thromboembolic event prophylaxis.

### Radiographic evaluation

**Fig. 2:**
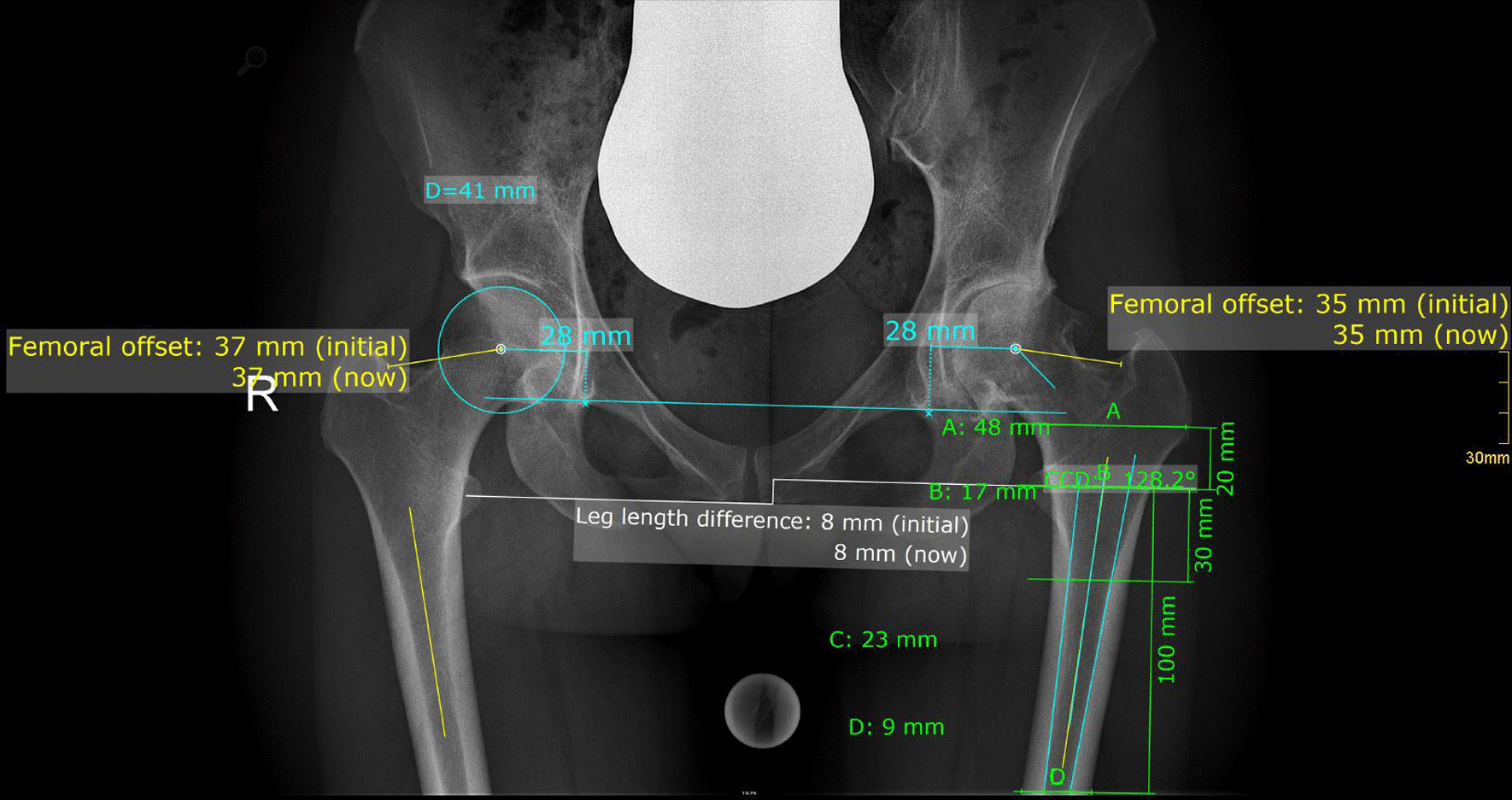
Preoperative measurements: Both sides: Femoral offset (FO), acetabular offset (AO), vertical position of the center of rotation (COR), leg length difference (LLD); affected hip: centrum–collum–diaphyseal angle (CCD angle), cortical index (CI), canal flare index, canal-to-calcar ratio

Radiographic measurement was performed on preoperative and 3 months postoperative digital low-centered AP radiographs of the pelvis [22]. Measurement was conducted independently by two reviewers (M.L. and C.S.), who were not involved in the index surgery. Radiographs were taken with the patient in standing position and with both legs in 15° internal rotation, and the central beam was directed on the symphysis pubis [23]. In order to achieve an accurate measurement of the hip anatomy, a double coordinate system was applied on both the preoperative and the postoperative images [24, 25]. Radiographic analysis was performed using MediCAD® Software V5.1 (Hectec GmbH, Altdorf, Germany). To characterize the anatomical shape of the proximal femur and the thickness of cortical bone, the canal-to-calcar ratio and the cortical index (CI) according to Dorr et al. [11] were determined. A high CI indicates a thick cortical bone [11]. Additionally, the canal flare index (CFI) according to Noble et al. [26] was determined. Radiographic leg length discrepancy (LLD) was measured as the perpendicular distance between line TT and the middle of the lesser trochanter (LT) [23]. The hip center of rotation (COR) was defined using a circle tool determining the diameter of the femoral head and its center [27]. The femoral offset (FO) was determined as the perpendicular distance between the COR and the proximal femoral shaft axis (FSA) [22, 27]. Acetabular offset (AO) was measured as the perpendicular distance between the COR and line T, with T being the perpendicular line on the transteardrop line (TT) through the ipsilateral teardrop figure [22]. Hip offset (HO) was calculated as the sum of FO and AO [22]. Centrum–collum–diaphyseal (CCD) angle was determined according to M. E. Müller on the affected hip [28]. The stem alignment was measured as the difference in degrees between the anatomic femoral shaft axis and the vertical stem axis [29]. The canal fill indices I, II and III (CFI I; CFI II; CFI III) were determined to evaluate the metaphyseal/diaphyseal filling of the femoral canal by the cementless stem implant on 3 different heights (CFI I: at the level of the LT, CFI II: 1 cm below the LT, and CFI III: 3 cm below the LT). On each height, the horizontal diameter of the stem implant was measured and divided by the endosteal medullary canal diameter, multiplied by 100 to achieve the relative percentage [16]. On preoperative X-rays, FO, AO, HO and LLD were measured bilaterally, while CCD angle, CI, canal flare index and canal-to-calcar ratio were measured unilaterally on the affected hip. Complete preoperative measurements are also shown in Fig. 2.

**Fig. 3:**
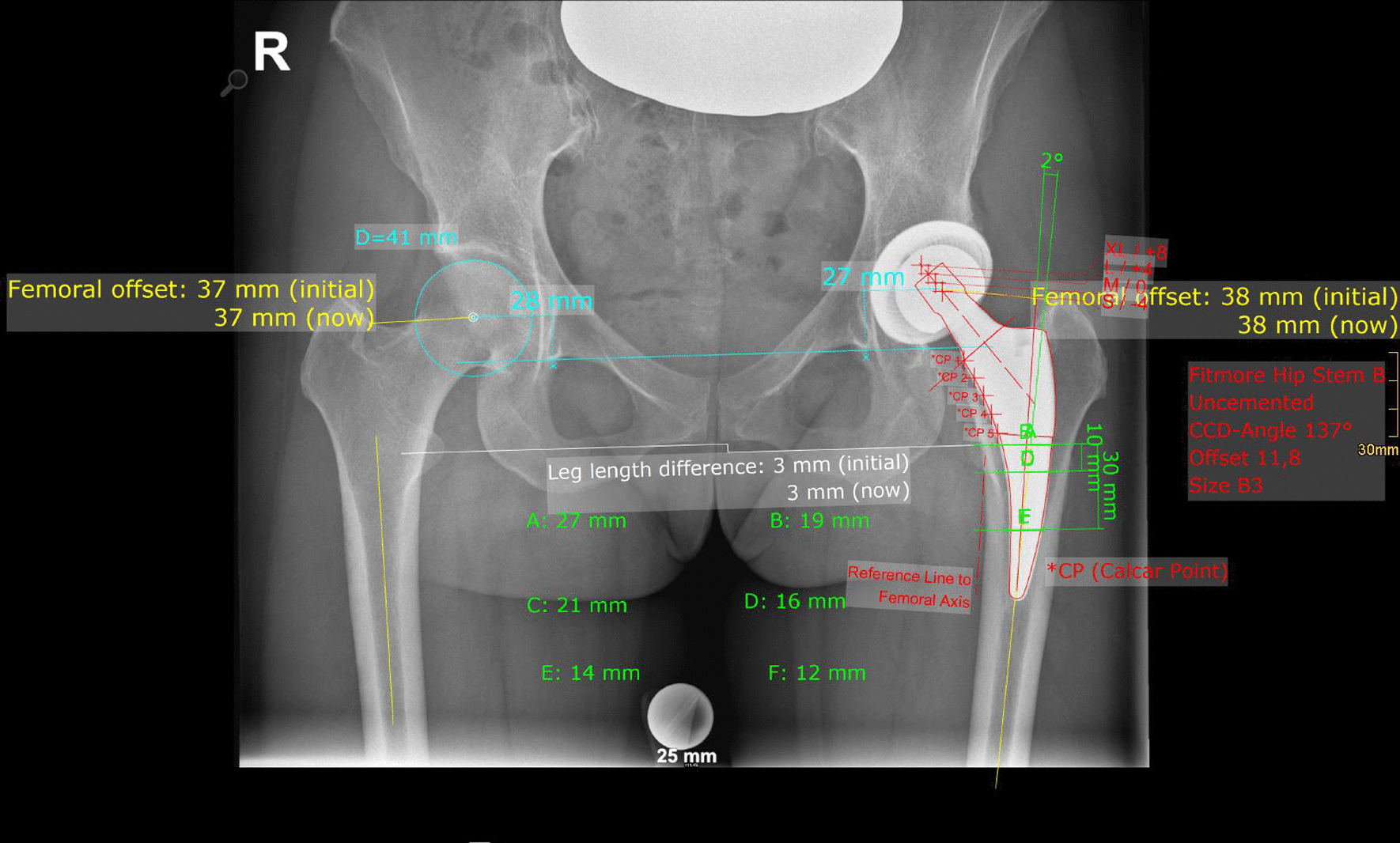
Postoperative measurements: Both sides: Femoral offset (FO), acetabular offset (AO), vertical position of the center of rotation (COR), leg length difference (LLD); affected side: stem alignment, canal fill indices I, II and III, cup inclination, cup anteversion

On postoperative X-rays, FO, AO, HO and LLD were measured bilaterally, and stem alignment, CFI I, CFI II and CFI III were measured unilaterally on the operated hip. Complete postoperative measurements are also shown in Fig. 3.

Intra- and interobserver reliabilities were calculated for 15 randomly selected cases for each group. Intraclass correlation coefficients (ICC) were used with a two-way random effects model for absolute agreement. Repeated measurements for intraobserver reliability were performed at day 1 and day 14 in a blinded fashion.

### Statistics

Descriptive statistical analysis was conducted for age, gender, body mass index (BMI) and laterality. A Shapiro–Wilk test was performed for testing for normal distribution. As not all variables were normally distributed, nonparametric testing was performed. For patient demographics, a Fisher’s exact test was performed on categorical variables (gender and side). A Kruskal–Wallis test was performed on continuous variables (age and BMI). For statistical analysis of pre- and postoperative radiographic measurements, a nonparametric Kruskal–Wallis test was performed. Power analysis was not performed due to the observed statistical significance [30]. The impact of the anatomical shape of the proximal femur on LLD and HO was evaluated with binary logistic regression models with the same confounding variables (Dorr type, canal flare index, cortical index, calcar-to-isthmus ratio, CCD angle, age, gender, BMI, surgeon’s experience, offset option, stem size and varus stem alignment). Binary output variables were defined according to the threshold value (LLD ≥ 5 mm; LLD ≥ 10 mm; HO ≥ 5 mm; HO ≥ 10 mm). Logistic regression for surgeon’s experience was evaluated by forming two groups: consultants; residents. Statistical analysis was calculated with SPSS version 27 (IBM SPSS statistics, Chicago, IL, USA). A *p* value < 0.05 was considered as statistically significant.

## Results

The overall interclass correlation coefficient between the 2 sets of measurements was 0.991% (95% confidence interval, 0.988–0.993, *p* < 0.001).

Patient demographics are shown in Table 1. Of the 106 included patients, 37 patients (34.9%) were addressed to Group A (Dorr type A), 59 patients (55.7%) to Group B (Dorr type B) and 10 patients (9.4%) to Group C (Dorr type C). There was no difference in patient demographics regarding age, BMI and operated side. The groups differed in gender distribution with significantly higher female predominance in Dorr types B and C (*p* < 0.001).

Preoperative measurements are shown in Table 1. The Dorr types were significantly different in canal flare index (*p* < 0.001), cortical index (*p* < 0.001) and canal-to-calcar ratio (*p* < 0.001). Dorr types A and B showed significantly lower CCD angles (*p* = 0.007) compared to Dorr type C. Postoperative measurements are also shown in Table 1. There was no significant difference detectable in all groups.

Logistic regression for LLD is shown in Table 2. For both thresholds (LLD ≥ 5 mm; LLD ≥ 10 mm), a significant risk was not detectable.

**Table 2:** Binary logistic regression for LLD ≥ 5 mm and LLD ≥ 10 mm

	LLD ≥ 5		LLD ≥ 10	
	Odds ratio (CI)	*P* value	Odds ratio (CI)	*P* value
Dorr type	0.543 (0.211–1.397)	0.205	3.711 (0.148–93.243)	0.425
Canal flare index	2.264 (0.634–8.086)	0.208	1.085 (0.29–41.101)	0.965
Cortical index	1.053 (0.361–3.065)	0.925	1.806 (0.131–25.002)	0.659
Canal-to-calcar ratio	0.711 (0.372–1.360)	0.302	0.299 (0.029–3.076)	0.310
CCD angle	0.987 (0.915–1.066)	0.742	0.888 (0.727–1.084)	0.243
Age	1.031 (0.990–1.074)	0.141	0.904 (0.757–1.079)	0.263
Gender	0.826 (0.283–2.409)	0.727	0.173 (0.004–8.358)	0.375
BMI	0.965 (0.882–1.056)	0.437	0.850 (0.604–1.197)	0.352
Surgeon’s experience	0.931 (0.389–2.226)	0.872	0.996 (0.046–21.329)	0.998
Offset option stem	1.484 (0.744–2.958)	0.262	0.137 (0.014–1.317)	0.085
Stem size	0.942 (0.730–1.216)	0.647	1.931 (0.833–4.477)	0.125
Varus stem alignment	0.395 (0.149–1.046)	0.061	1.994 (0.146–27.263)	0.605

Logistic regression for HO is shown in Table 3. Using a higher offset option was a risk factor for an increased HO ≥ 5 mm compared to the contralateral healthy hip (OR = 2.252; CI: 1.069–4.745; *p* = 0.033). For all other parameters, a significantly increased risk was not detectable. For the threshold LLD ≥ 10 mm, there were no significant differences detectable for all parameters tested in logistic regression.

**Table 3:** Binary logistic regression for HO ≥ 5 mm and HO ≥ 10 mm

	HO ≥ 5		HO ≥ 10	
	Odds ratio (CI)	*P* value	Odds ratio (CI)	*P* value
Dorr type	1.497 (0.566–3.959)	0.417	1.875 (0.414–8.493)	0.415
Canal flare index	0.390 (0.105–1.448)	0.160	0.381 (0.057–2.573)	0.322
Cortical index	1.703 (0.559–5.190)	0.349	1.219 (0.238–6.242)	0.812
Canal–calcar ratio	1.364 (0.698–2.666)	0.363	1.114 (0.392–3.163)	0.840
CCD angle	0.981 (0.904–1.064)	0.640	0.981 (0.874–1.101)	0.745
Age	1.019 (0.976–1.064)	0.396	1.009 (0.946–1.077)	0.780
Gender	0.951 (0.307–2.939)	0.930	1.018 (0.204–5.088)	0.983
BMI	0.994 (0.906–1.091)	0.902	0.970 (0.834–1.127)	0.689
Surgeon’s experience	0.729 (0.287–1.854)	0.507	0.565 (0.109–2.929)	0.496
Offset option stem	2.252 (1.069–4.745)	**0.033**	1.546 (0.487–4.903)	0.460
Stem size	0.989 (0.754–1.298)	0.936	1.048 (0.694–1.581)	0.825
Varus stem alignment	1.378 (0.506–3.758)	0.531	2.759 (0.507–15.000)	0.240

Bold value signal statistically significant values in testing

## Discussion

The morphology of the proximal femur was not identified as a risk factor for a LLD ≥ 5 mm and ≥ 10 mm as well as an increase of HO ≥ 5 mm and ≥ 10 mm in short-stem THA. The femoral shape according to Dorr classification as well as CFI, CI and canal-to-calcar ratio did not pose an increased for risk for a LLD and an increased HO above 5 mm or 10 mm compared to a contralateral healthy hip.

Postoperative LLD can affect the functional outcome after THA adversely [10]. A LLD ≤ 5 mm and an increase in HO ≤ 5 mm are seen to be beneficial for postoperative clinical outcome in cementless THA [8]. Postoperative clinical outcome decreases with every 5 mm increase in HO and LLD [8]. Additionally, a LLD greater than 7–10 mm is often perceived by patients [31]. Brumat et al. [13] detected higher CFI as a risk factor for LLD ≥ 5 mm with an odds ratio of 4.5 (*p* = 0.03) in cementless THA with metaphyseal fixation, while diaphyseal and cemented fixation did not show an increased risk. CFI was not detected as a risk factor in the presented study in cementless short-stem THA for HO and LLD. Lim et al. [12] report the risk of leg length increase in Dorr type A femurs and the risk of leg length decrease in Dorr type C femurs in cementless straight-stem THA. A higher cortical index (CI) as found in Dorr type A femurs shows a significantly higher LLD (*p* = 0.003) [12]. We did not detect a significant difference in LLD depending on Dorr type (*p* = 0.532) in short-stem THA. Also, logistic regression did not show any significantly increased risk for LLD depending on Dorr type or CI. Testing for differences in LLD as well as logistic regression for Dorr type, CFI, CI and canal-to-calcar ratio did not show differences or a significantly increased risk for a LLD ≥ 5 mm or ≥ 10 mm. Therefore, we conclude that the shape of the proximal femur does not pose a risk for LLD in cementless short-stem THA with a meta-diaphyseal short stem.

In order to evaluate possible confounder, we also carried out the logistic regression for other variables such as age, gender or BMI. Warnock et al. [32] detected a lengthening in cementless THA in females due to a greater femoral height discrepancy leading to reduced delta gain in Oxford hip score (OHS) and pain scores. Al-Amiry et al. [33] detected a negative effect of increased BMI in restoration of leg length but not on restoration of femoral offset. In our study, we could not detect a significantly increased risk for increased LLD and HO for gender and BMI. Besides patient demographics, we also evaluated offset option, size of the implanted stem and varus stem alignment as possible confounder. We could not detect any increased risk for increased postoperative LLD. We could only detect a significant risk for increased HO ≥ 5 mm by using higher offset options.

Surgeon’s experience is also a confounding factor in maintaining leg length in THA. Kishimoto et al. [34] found out that 80% of patients operated by high-volume surgeons had a LLD < 5 mm compared to 40% of patients operated by low-volume surgeons. Low-volume surgeons are a risk factor for increased postoperative LLD with an odds ratio of 8.26 [34]. We evaluated LLD and reconstruction of HO according to surgeon’s experience by evaluating differences between consultants and residents. We did not find a significantly increased risk for increased postoperative LLD or increased HO in THA performed by less experienced surgeons.

We also evaluated stem sizing and implant positioning. Stem alignment and canal fill indices were without significant difference in all Dorr types. Apart from that, high CFI is also seen as a risk factor of femoral component undersizing, particularly with taper wedge stems, due to potting the stem distally in the narrow canal. We could not detect a negative on stem sizes without any statistical significance for canal fill indices [35]. A canal fill index < 80% is seen as undersized in cementless straight-stem THA [35]. Comparable canal fill indices for Fitmore® hip stem show higher values with a canal fill indices between 85.2% and 90.4% [16]. We report lower values between 76.7 and 84.6%. However, the anatomical shape of the proximal femur was not a risk factor for implant undersizing in the presented study. The generally lower canal fill indices are more likely to be a result of less aggressive broaching and accepting lower canal fill. The long-term effect of lower canal fill indices for short stems has not been evaluated fully. However, the anatomical shape of the proximal shape does not pose a risk factor for implant undersizing of the femoral component.

Several limitations of the study have to be addressed. Firstly, we tried to minimize a potential selection bias with very strict inclusion criteria. Only patients with a single implant design and approach were included in this study. A homogenous study cohort was created by excluding patients with a bilateral hip disease (Kellgren–Lawrence > grade 1). Apart from gender distribution, there were not any significant differences between the three different study groups for age at surgery, BMI or side. Also, measurements of preoperative differences in FO, AO and HO as well as LLD were carried out without any significance. Furthermore, we aimed to increase reliability of the measurements and results by restricting inclusion based on preoperative diagnosis. We excluded all forms of secondary osteoarthritis of the hip and development dysplasia of the hip Crowe grade II to IV. Prior surgery before THA was also excluded. However, mild hip dysplasia (lateral center–edge angle 20°–25°), coxa profunda and morphologic alterations related to cam- or pincer-type impingement were included, because these changes might be subtle and cannot be reliably identified in the present study cohort with end-stage disease. Therefore, we conclude that the findings in the present study are applicable for primary osteoarthritis and care must be taken when applying our findings on secondary osteoarthritis or high grades of development dysplasia of the hip. Secondly, we address the fact of taking measurements on plain radiographs. FO is underestimated by approximately 13% on plain radiographs [27]. Additionally, radiographic measurement of leg length difference does not necessarily reflect clinical leg length difference [36]. Additionally, in radiographic measurements the potential disadvantage of malpositioning the patient in the X-ray beam or malrotation of the pelvis and femur is potential factors for disadvantage in accuracy. However, our measurements are easily reproducible, applicable in daily routine and less invasive regarding radiation exposure. Furthermore, we postulate variances in inter- and intraobserver reliability in measuring clinical leg length difference.

## Conclusion

The anatomical shape of the proximal femur has no negative influence on the reconstruction of hip offset and leg length difference in cementless total hip arthroplasty with a curved short stem with meta-diaphyseal fixation. Offset reconstruction and leg length difference are not negatively influenced by Dorr type, canal flare index, cortical index and canal-to-calcar ratio. Additionally, the morphology of the proximal femur does not lead to stem undersizing or higher varus stem positioning.

## Data Availability

Data and materials are available on request.

## Abbreviations

HO: Hip offset
THA: Total hip arthroplasty
LLD: Leg length difference
HHS: Harris hip score
CFI: Canal Flare Index
AP: Anteroposterior
BMI: Body mass index
CI: Cortical index
LT: Lesser trochanter
COR: Center of rotation
FO: Femoral offset
FSA: Femoral shaft axis
AO: Acetabular offset
TT: Transteardrop line
CCD: Centrum–collum–diaphyseal
CFI I; CFI II; CFI III: Canal fill indices I, II and III
ICC: Intraclass correlation coefficients
OR: Odds ratio
OHS: Oxford hip score
